# Protective Effect of Silymarin Against Paclitaxel‐Induced Cardiotoxicity

**DOI:** 10.1002/fsn3.71710

**Published:** 2026-04-02

**Authors:** Tuğçe Tutar, Seda Yakut, Adem Kara, Aykut Ulucan, Cüneyt Çağlayan

**Affiliations:** ^1^ Department of Physiology, Faculty of Veterinary Medicine Bingöl University Bingöl Türkiye; ^2^ Department of Histology and Embryology, Faculty of Veterinary Medicine Burdur Mehmet Akif Ersoy University Burdur Türkiye; ^3^ Department of Molecular Biology and Genetics, Faculty of Science Erzurum Technical University Erzurum Türkiye; ^4^ Department of Medical Services and Techniques, Vocational School of Health Services Bingöl University Bingöl Türkiye; ^5^ Department of Medical Biochemistry, Faculty of Medicine Bilecik Şeyh Edebali University Bilecik Türkiye

**Keywords:** apoptosis, cardiotoxicity, inflammation, oxidative stress, paclitaxel, silymarin

## Abstract

Paclitaxel, a widely used chemotherapeutic agent, is known for its efficacy against various cancers but is also associated with significant cardiotoxicity. This study aimed to explore the potential of silymarin in mitigating Paclitaxel‐induced cardiotoxicity in rats. To establish a model of Paclitaxel‐induced cardiotoxicity, rats were injected intraperitoneally with Paclitaxel at 2 mg/kg for five consecutive days. Additionally, starting from day 6, silymarin was administered orally at a dose of 200 mg/kg for 10 days. Our findings indicate that silymarin reduced malondialdehyde (MDA) levels and increased the levels of antioxidant enzymes (SOD, CAT, GPx, and GSH) in heart tissue. Additionally, silymarin reduced serum cytokine levels (IL‐1β, TNF‐α, and IL‐6) and improved serum lipid profiles, reducing lactate dehydrogenase (LDH), high‐density lipoprotein (HDL), low‐density lipoprotein (LDL), triglyceride, and total cholesterol levels. Histopathological analysis revealed reduced inflammatory reaction scores in heart tissues. Immunohistochemical analysis revealed decreased PI3 kinase p85α scores, and western blot analysis showed downregulation of P2X7R, Interleukin‐1β (IL‐1β), Tumor Necrosis Factor‐α (TNF‐α), and nuclear factor kappa B p65 (NF‐κB‐p65) expression. Silymarin also reduced Caspase‐3 levels while increasing Bcl‐2 protein expression, an antiapoptotic protein. These results suggest that silymarin has significant therapeutic potential for reducing Paclitaxel‐induced cardiotoxicity via its antioxidant, anti‐inflammatory, and anti‐apoptotic properties.

## Graphical abstract

- Paclitaxel, a potent chemotherapeutic agent, induces oxidative stress, inflammation, and apoptosis leading to cardiotoxicity.
- In this study, silymarin supplementation significantly reduced oxidative stress markers, downregulated inflammatory cytokines (IL‐1β, TNF‐α, NF‐κB), and restored the balance between pro‐ and anti‐apoptotic proteins in rat heart tissue.
- These findings highlight silymarin's antioxidant, anti‐inflammatory, and anti‐apoptotic properties as a promising protective approach against chemotherapy‐induced cardiotoxicity.

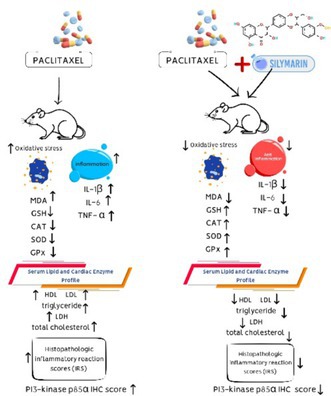

## 1 Introduction

Paclitaxel, derived from *Taxus brevifolia* , is a potent cytotoxic agent used since the 1990s for ovarian and breast cancers and later for other cancers, such as lung, bladder, colon, and esophageal cancers (Gao et al. 2024). Its broad antineoplastic activity is accompanied by dose‐limiting toxicities, including cardiovascular adverse effects that remain clinically relevant (Balachandran et al. 2024; Sati et al. 2024).

Beyond overt events, cancer‐therapy–related cardiac dysfunction (CTRCD) can be subclinical and detectable only with sensitive imaging. Network meta‐analysis shows that myocardial strain (particularly global longitudinal strain) detects early CTRCD across anticancer regimens, supporting its use for surveillance (Dong et al. 2025). Contemporary cardio‐oncology guidelines also emphasize oxidative stress and inflammation as central mechanisms of CTRCD and highlight multimodal prevention strategies (Contaldi et al. 2025). Inflammation is increasingly recognized as a mechanistic driver of chemotherapy‐induced cardiotoxicity, with NF‐κB signaling and inflammasome activation converging on downstream cytokines (e.g., TNF‐α and IL‐1β) (Hutchins et al. 2024). In the heart, purinergic P2X7 receptor signaling can amplify the NLRP3/IL‐1β pathway during stress and disease, providing a plausible upstream trigger for inflammatory cardiotoxicity (Higashikuni et al. 2023; Shao et al. 2024).

Paclitaxel can induce subclinical cardiotoxicity, as evidenced by significant decreases in left ventricular strain measures in patients treated with Paclitaxel plus carboplatin, despite no clinical heart failure symptoms (Kanar et al. 2022). Paclitaxel‐induced oxidative stress, associated with increased reactive oxygen species (ROS) and decreased antioxidant levels (Glutathione (GSH), Superoxide Dismutase (SOD), and Glutathione Peroxidase (GPx)), contributes to cardiotoxicity (Khaled et al. 2022; Meshkini and Yazdanparast 2012).

Since natural antioxidants reduce oxidative stress, modulate inflammatory pathways, and strengthen endogenous antioxidant defenses, their therapeutic potential in mitigating such side effects is an expanding field of study (Khaled et al. 2022; Şengül et al. 2017). With increasing evidence of their cardioprotective potential, natural antioxidants are being investigated as adjuncts to reduce CTRCD by inhibiting oxidative and inflammatory signaling (Cadeddu Dessalvi et al. 2021; Marino et al. 2023). Silymarin, a flavonolignan complex derived from *Silybum marianum* , has been shown to exhibit antioxidant and anti‐inflammatory properties in both preclinical and clinical settings. It also inhibits NF‐κB activation (Zhao et al. 2024). The current study was designed to assess the cardioprotective potential of silymarin against paclitaxel‐induced cardiac injury. Our specific goals were to determine whether silymarin could reduce oxidative stress, inhibit inflammatory signaling mediated by P2X7R/NF‐κB, and rebalance pro‐ and anti‐apoptotic proteins. By lowering oxidative stress and inflammation and inhibiting apoptosis through modification of the P2X7R/NF‐κB signaling axis, we predicted that silymarin would reduce paclitaxel‐induced cardiotoxicity.

## 2 Materials and Methods

### 2.1 Chemicals

Paclitaxel (Taxol) was obtained from Koçak Farma, Istanbul, Turkey, and Silymarin (purified compound) from Sigma‐Aldrich, St. Louis, MO. All other chemicals were of analytical grade and purchased from Sigma‐Aldrich.

### 2.2 Experimental Procedure

Male albino Wistar rats (12 weeks old, 250–300 g) were used in this study. Animals were randomly assigned into four experimental groups, each consisting of 7 rats (*n* = 7/group): Control (physiological saline), SIL (silymarin 200 mg/kg), PAX (paclitaxel 2 mg/kg), and PAX+SIL (paclitaxel 2 mg/kg + silymarin 200 mg/kg). Paclitaxel was administered intraperitoneally at 2 mg/kg (Polomano et al. 2001) for 5 days. Silymarin administration (200 mg/kg, oral) (Yakut et al. 2024, 2025) began on the sixth day and continued for 10 days after Paclitaxel treatment had concluded. Ethical approval was granted by the Animal Experiments Ethics Committee of Bingöl University (Document date and number: 28.06.2021–E.20008). The study groups and procedures are summarized in Table 1.

**TABLE 1:** Experimental groups and experimental procedure.

Groups	0. day	5. day	Euthanasia/Sacrifice
Control (*n* = 7)	—	Physiological saline was given by oral gavage.	Animals were sacrificed on the 15th day of the study.
SIL (*n* = 7)	—	Silymarin at a dose of 200 mg/kg was administered by oral gavage for 10 days.	Animals were sacrificed on the 15th day of the study.
PAX (*n* = 7)	Paclitaxel was given intraperitoneally at a dose of 2 mg/kg for 5 days.	Physiological saline was given by oral gavage for 10 days.	Animals were sacrificed on the 15th day of the study.
PAX + SIL (*n* = 7)	Paclitaxel was given intraperitoneally at a dose of 2 mg/kg for 5 days.	Silymarin at a dose of 200 mg/kg was administered by oral gavage for 10 days.	Animals were sacrificed on the 15th day of the study.

### 2.3 Biochemical Analysis

Heart tissues were homogenized, and oxidative stress markers (malondialdehyde (MDA), GSH, SOD, catalase (CAT), and GPx) were measured (Aebi 1984; Matkovics 1988; Placer et al. 1966; Sedlak and Lindsay 1968; Sun et al. 1988). Serum cytokine concentrations (IL‐1β, TNF‐α, and IL‐6) were measured using rat‐specific ELISA kits (SunRed Biological Technology, Shanghai, China; IL‐1β: Cat. No. 201–11‐0120, TNF‐α: 201–11‐0765, IL‐6: 201–11‐0136). According to the manufacturer's validation data, the assay ranges were 15–3000 ng/L for IL‐1β, 8–1000 ng/L for TNF‐α, and 2–600 pg/mL for IL‐6, with minimum detectable concentrations of 10.135 ng/L, 5.127 ng/L, and 1.822 pg/mL, respectively. The intraassay and interassay coefficients of variation for all kits were < 10% and < 12%, indicating acceptable assay precision. Lipid profiles (LDH, HDL, LDL, triglycerides, and total cholesterol) were measured using an autoanalyzer (Toshiba, Japan).

### 2.4 Histopathology and Immunohistochemistry

Heart tissues were fixed, sectioned, and stained for histopathological examinations. The inflammatory reaction score (IRS) was used to assess myocardial inflammation (Wang, Zhang, et al. 2017). Immunohistochemical analysis was performed using an anti‐PI3‐kinase p85α antibody to determine the staining intensity in heart tissues. IRS was evaluated independently by two blinded histologists, without knowledge of group allocation.

### 2.5 Western Blot Analysis

Heart tissues were processed for western blot analysis to measure the protein expression levels (Caspase‐3, Bcl‐2, nuclear factor kappa B p65 (NF‐κB‐p65), IL‐1β, TNF‐α, P2X7R, and beta‐actin). The samples were weighed and then pulverized using liquid nitrogen. The specimens were processed using RIPA buffer supplemented with inhibitors of both proteases and phosphatases. Tissue homogenization was conducted using the Qiagen Tissue Lyser System at 30 Hz for 20 s. A protein assay kit (Pierce BCA, Thermo Scientific, USA) was employed to determine the total protein content in the heart tissue. SDS‐PAGE (10%) was used to separate 30 μg of total protein by molecular weight, and the proteins were then transferred to PVDF membranes. The membranes were blocked with 5% w/v bovine serum albumin for 90 min. Primary antibodies (IL‐1β [sc‐52,012], TNF‐α [sc‐52,746], Caspase‐3 [sc‐56,053], Bcl‐2 [sc‐7382], NF‐κB‐p65 [sc‐109], and beta‐actin [sc‐47,778], Santa Cruz Biotechnology, USA; P2X7R [11144–1‐AP], Proteintech Group, USA) were applied to the membranes and incubated at 4°C for approximately 15 h. Following a wash with Tris‐buffered saline containing Tween, the membranes were exposed to secondary antibodies (sc‐2004/sc‐2005, Santa Cruz Biotechnology, USA) conjugated with horseradish peroxidase (TP‐125‐HL, Thermo Fisher Scientific, USA) for 90 min. β‐actin (1:2000 dilution) was used as the loading control. Protein bands were visualized using enhanced Western ECL substrate (3405, Thermo Fisher Scientific, USA) and analyzed with software (Image Lab, Bio‐Rad, USA).

### 2.6 Statistical Analysis

The data were analyzed using Minitab 16.2.0.0 software. One‐way ANOVA and post hoc Tukey tests were used to determine group differences, with significance set at *p* < 0.05.

## 3 Results

### 3.1 Paclitaxel Induction Elicits Severe Oxidative Stress and Dyslipidemia in Cardiac Tissue, Which Can Be Mitigated by Silymarin Administration

Paclitaxel increased MDA levels and decreased GSH, CAT, GPx, and SOD levels in the heart tissues, whereas silymarin treatment reversed these changes (*p* < 0.05). The MDA, GSH, CAT, GPx, and SOD levels, which are oxidative stress parameters in heart tissue, are presented in Table 2. Serum cytokine levels (IL‐1β, TNF‐α, and IL‐6) were significantly higher in the Paclitaxel group but decreased with silymarin treatment (*p* < 0.05). The serum levels of IL‐1β, TNF‐α, and IL‐6 are shown in Table 2.

**TABLE 2:** Levels of oxidative stress markers and IL‐1β, TNF‐α, and IL‐6 levels.

Parameters	Control	SIL	PAX	PAX + SIL
MDA (nmol/g tissue)	48.31 ± 3.29^a^	46.42 ± 3.04^a^	65.45 ± 1.44^c^	53.12 ± 2.48^b^
GSH (nmol/g tissue)	1.34 ± 0.08^c^	1.37 ± 0.06^c^	0.89 ± 0.08^a^	1.14 ± 0.09^b^
CAT (katal/g protein)	32.82 ± 1.80^c^	34.08 ± 1.83^c^	20.47 ± 1.24^a^	26.65 ± 1.17^b^
SOD (U/g protein)	18.94 ± 1.54^c^	19.69 ± 1.59^c^	12.38 ± 1.09^a^	15.29 ± 0.77^b^
GPx (U/g protein)	25.76 ± 1.30^c^	27.00 ± 1.74^c^	16.61 ± 1.19^a^	20.10 ± 1.23^b^
IL‐1β (pg/L)	910.17 ± 88.32^a^	907.25 ± 73.56^a^	1294.09 ± 97.71^c^	1057.63 ± 71.19^b^
IL‐6 (pg/mL)	35.65 ± 4.51^a^	36.63 ± 4.96^a^	49.06 ± 4.42^c^	42.67 ± 4.08^b^
TNF‐ α (ng/ml)	93.16 ± 15.18^a^	94.82 ± 12.96^a^	148.74 ± 17.08^c^	112.81 ± 10.71^b^

*Note:* Different superscripts (a–d) in the same row indicate significant differences (*p* < 0.05) between the groups.

**FIGURE 1:**
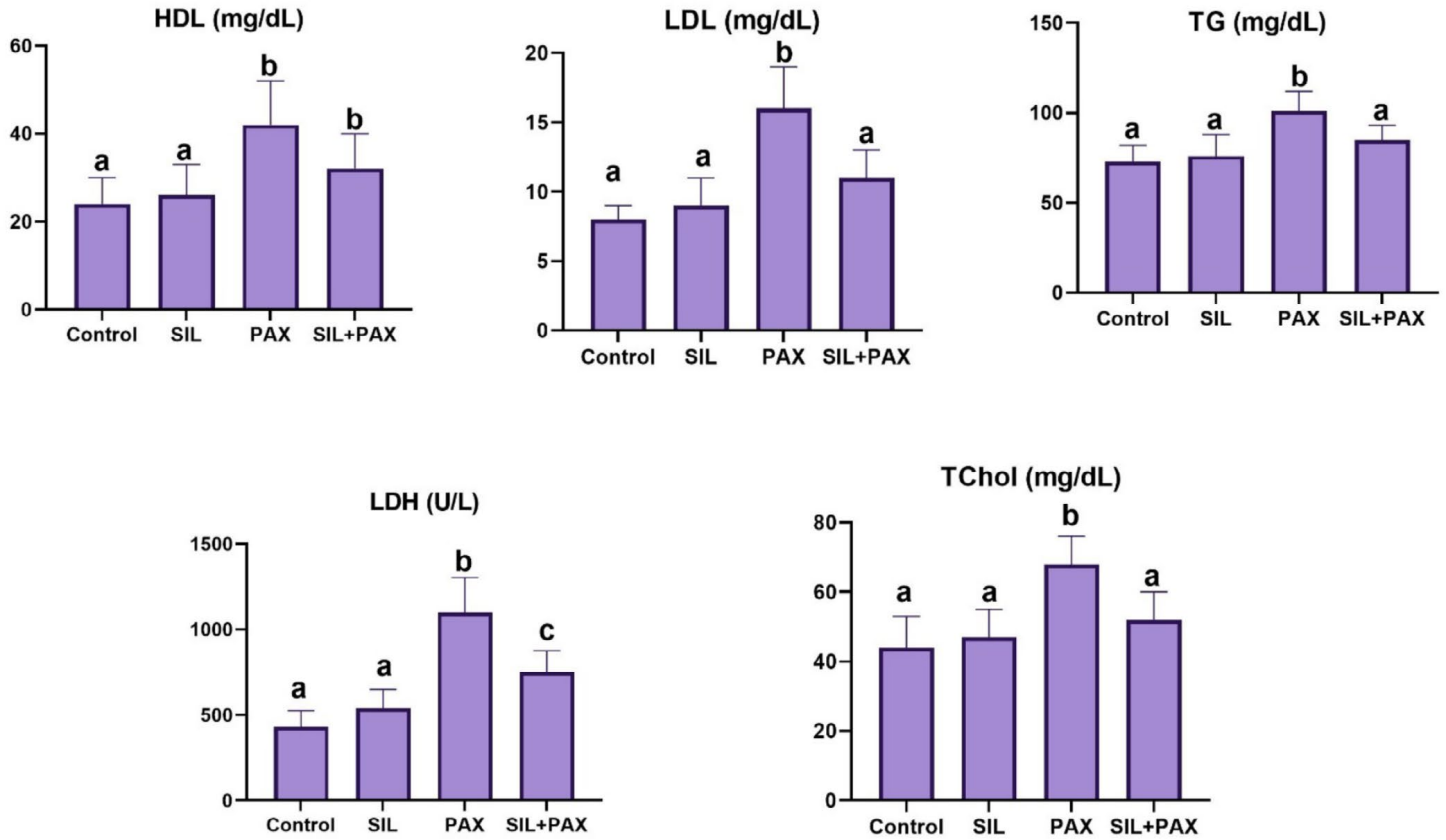
Serum HDL, LDL, triglyceride, LDH, and total cholesterol values of the groups (different superscripts (a–c) indicate a significant difference (*p* < 0.05) between groups).

Similarly, Paclitaxel increased serum LDH, HDL, LDL, triglyceride, and total cholesterol levels, which were reduced by silymarin (*p* < 0.05). The serum LDH, HDL, LDL, triglyceride, and total cholesterol levels are shown in Figure 1.

### 3.2 Silymarin Reduces Paclitaxel‐Induced Cardiac Tissue Damage and Myofibrillar Degeneration

Histopathological examination revealed that silymarin reduced paclitaxel‐induced cardiac tissue damage, as indicated by lower IRS scores in the silymarin‐treated group (*p* < 0.05). IRS was negative in both the control and SIL groups. The IRS scores for each group are shown in Table 3.

**TABLE 3:** Histopathologic inflammatory reaction scores (IRS) of the experimental groups.

Group	IRS
CONTROL	0.00 ± 0.00
SIL	0.00 ± 0.00
PAX	3.00 ± 0.43^a^
PAX+SIL	1.76 ± 0.46^a^, ^b^

^a^ *p* < 0.05 Compared to CONTROL and SIL.

^b^ *p* < 0.05 compared with PAX.

**FIGURE 2:**
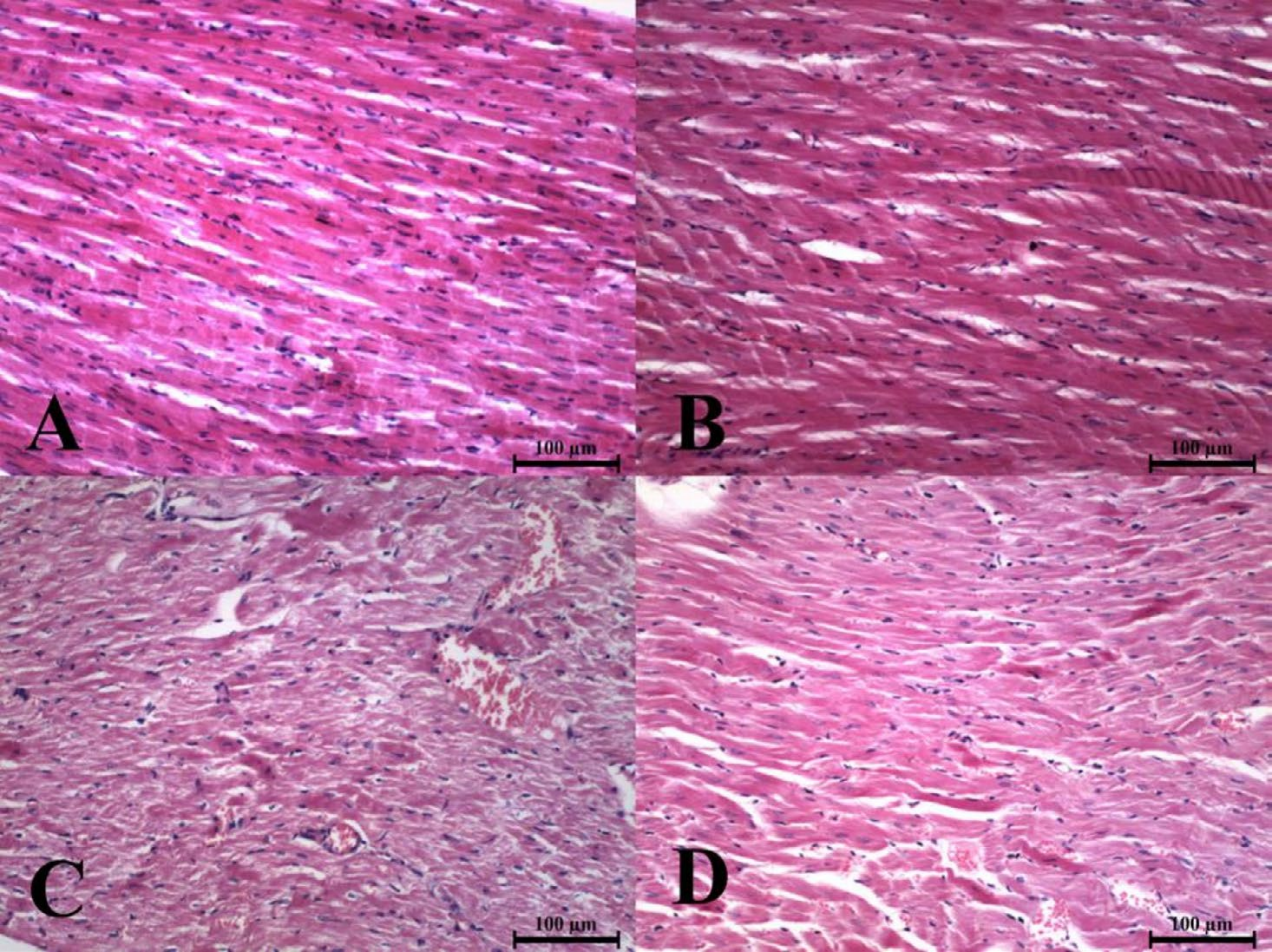
Histopathological image of heart tissues of experimental groups, H&E staining, 200× magnification. Experimental groups: (A) Control (IRS = 0), (B) SIL (IRS = 0), (C) PAX (IRS = 4), and (D) PAX+SIL (IRS = 2).

It was observed that the histological architecture of the myocardium was preserved in the control (Figure 2A) and SIL (Figure 2B) groups, and no pathological findings were detected. In addition, the PAX group exhibited myofibrils with perinuclear sarcomeric degeneration (myolysis), basophilic myofibrillar degeneration, myocytes with dark pyknotic nuclei, focal fibroblasts, and dilated capillaries (Figure 2C). In the PAX+SIL group, these findings were significantly reduced (*p* < 0.05) (Figure 2D).

### 3.3 Silymarin Modulates PI3K, Inflammatory Pathway Proteins, and Apoptotic Factors in Paclitaxel‐Induced Cardiac Injury

**FIGURE 3:**
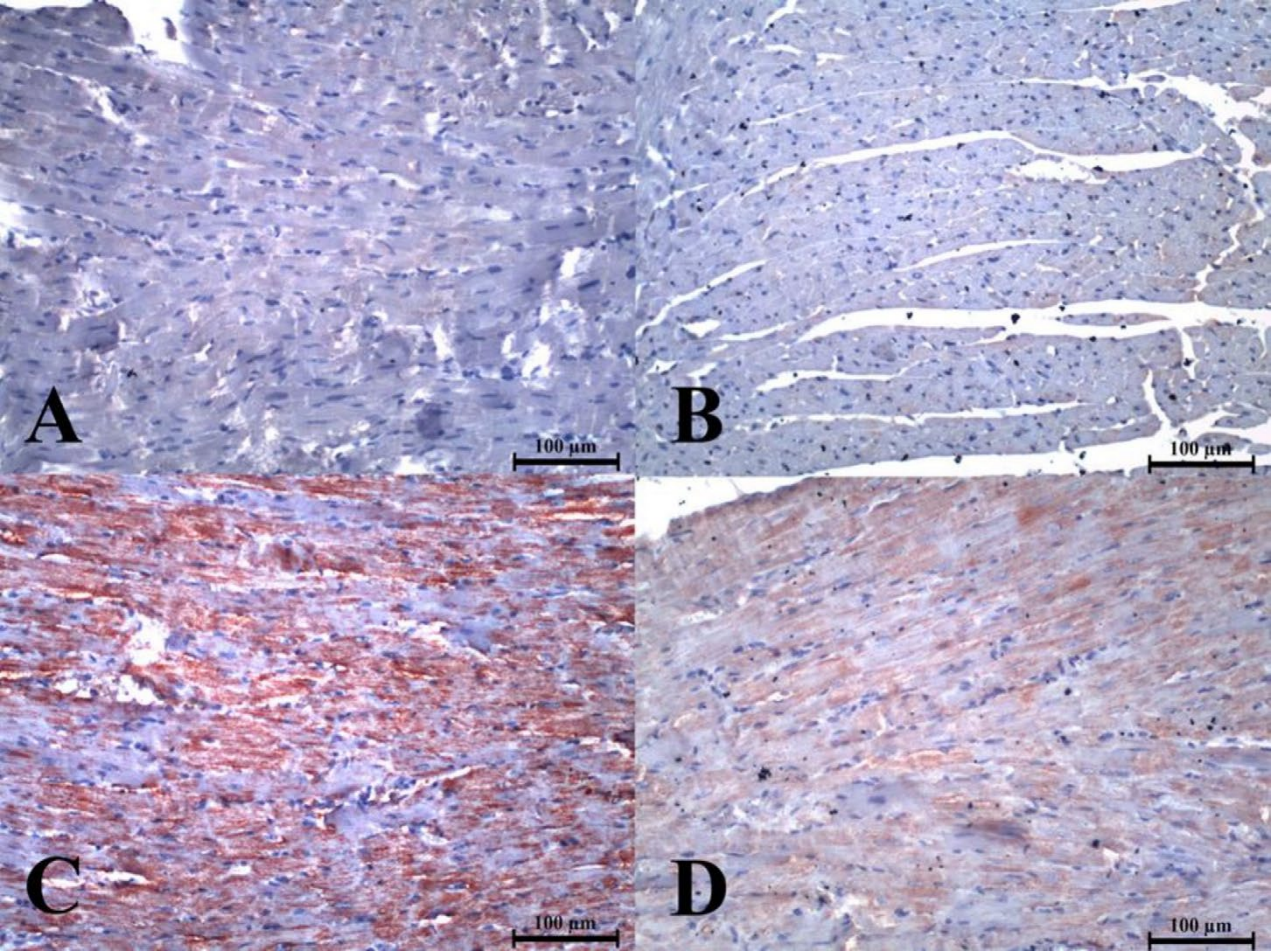
IHC staining image of heart tissues of experimental groups with anti‐PI3‐kinase p85α antibody, IHC staining, 200× magnification. Experimental groups: (A) Control (IHC score = 0), (B) SIL (IHC score = 0), (C) PAX (IHC score = 3), and (D) PAX+SIL (IHC score = 2).

Immunohistochemical analysis revealed decreased PI3 kinase p85α staining in the silymarin‐treated group (*p* < 0.05). The IHC staining score in the PAX+SIL group was significantly lower than that in the PAX group (*p* < 0.05) (Figure 3).

The IHC staining scores for anti‐PI3‐kinase p85α antibodies are shown in Table 4.

**TABLE 4:** Immunohistochemical (IHC) staining scores of the experimental groups.

Group	IHC score
CONTROL	0.38 ± 0.40
SIL	0.80 ± 0.18
PAX	2.76 ± 0.25^a^
PAX+SIL	1.57 ± 0.57^a^, ^b^

^a^ *p* < 0.05 compared with CONTROL and SIL.

^b^ *p* < 0.05 compared with PAX.

Western blot analysis revealed reduced expression of P2X7R, NF‐κB‐p65, IL‐1β, TNF‐α, and Caspase‐3, and increased Bcl‐2 levels in the silymarin‐treated group compared to the PAX group (*p* < 0.05).

**FIGURE 4:**
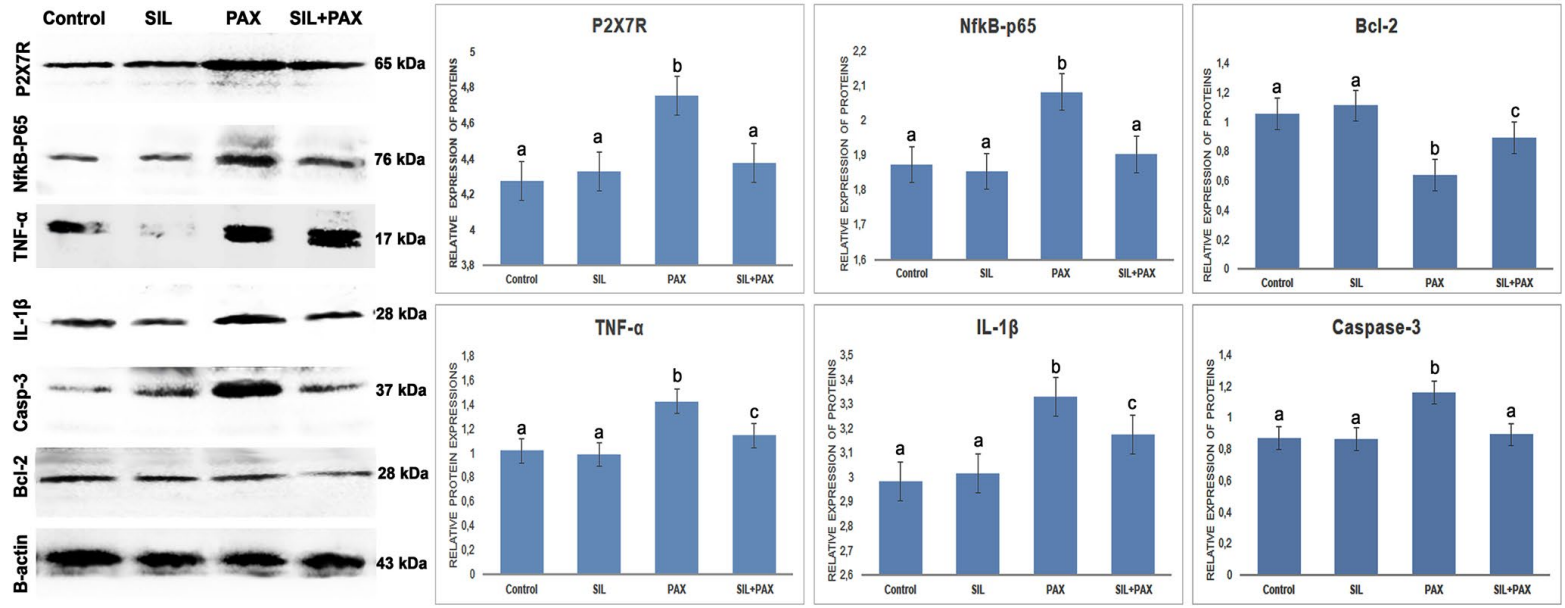
Relative protein expression of P2X7R, NfkB‐p65, Bcl‐2, TNF‐α, IL‐1β, and Caspase‐3 in rat heart tissues. Different superscripts (a–c) in the same row indicate a significant difference (*p* < 0.05) between the groups.

The protein expression levels and comparisons between the groups are shown in Figure 4.

## 4 Discussion

Paclitaxel, a widely used antineoplastic agent, is known to cause cardiotoxicity. It exerts its therapeutic action by binding to β‐tubulin, promoting microtubule polymerization, and halting cell division by inhibiting mitotic activity (Al‐Mahayri et al. 2021). The future use of a drug to cause CTRCD is considered mainly determined by how it affects the heart and regulates calcium inside the cell. It is believed that a part of the mechanism of doxorubicin cardiotoxicity lies in the disruption of intracellular calcium regulation, mitochondrial dysfunction, and oxidative stress. The drug causes heart cell death and contractile dysfunction by acting on the sarcoplasmic reticulum, disrupting calcium homeostasis (Chang and Wang 2018). In addition, the oxidative stress caused by paclitaxel may be one of the factors contributing to cardiac contractile dysfunction, together with structural damage to myofibrils (Pentassuglia et al. 2007; Varricchi et al. 2018).

Khaled et al. (2022) declare that paclitaxel cardiotoxicity is an oxidative stress‐related condition that leads to the formation of MDA as well as the inhibition of antioxidant MDA and the consequent drop in enzyme activities like SOD, CAT, and GPx. In the heart tissues of animals treated with Paclitaxel, there were also higher levels of MDA and lower levels of GSH, SOD, CAT, and GPx. Silymarin is a potent antioxidant that has been used to treat several cardiac ailments. Besides, its role in reducing oxidative stress and enhancing endogenous antioxidant enzyme activities in drug‐induced cardiotoxicity has been claimed to include doxorubicin (İpek and Tunca 2023). The present study reveals that the mechanism of the paclitaxel‐induced oxidative stress protection is the lowering of MDA levels and the raising of GSH, SOD, CAT, and GPx activities. Oxidative stress undermines the heart cells, making them undergo lipid peroxidation and protein oxidation (Khaled et al. 2022). On the other hand, the antioxidant properties of silymarin act as a shield against such damage, while its free radical‐scavenging properties are a significant factor in its ability to counteract the oxidative stress induced by Paclitaxel. Interestingly, these factors were comparable only to those of the control group in the silymarin group, indicating that it does not disrupt oxidative balance under normal physiological conditions. It indicates that silymarin is nontoxic under standard conditions, and its cardio‐protective effect occurs only under pathological stress.

Proinflammatory cytokines like IL‐1β, TNF‐α, and IL‐6 are pivotal in mediating inflammatory responses. Elevated levels of these factors contribute to oxidative stress, endothelial dysfunction, and cardiomyocyte apoptosis, which are key mechanisms underlying cardiotoxicity induced by various chemotherapeutic agents (Birari et al. 2020; Wang et al. 2019). This study showed that Paclitaxel exposure increased serum levels of these cytokines, possibly reflecting impaired cell‐mediated immune response. Overall, the anti‐inflammatory effects of silymarin are due to the inhibition of the NF‐κB signaling pathway, which is responsible for the production of pro‐inflammatory cytokines such as IL‐1β, TNF‐α, and IL‐6. Meanwhile, recent in vivo studies have revealed reductions in NF‐κB expression and subsequent cytokine generation by silymarin in models of liver injury (El‐Kot et al. 2023), while comprehensive reviews argue for a broader set of anti‐inflammatory mechanisms of silymarin in numerous disease settings (Surai et al. 2024; Zhao et al. 2024). Therefore, suppression of this pathway reduces Paclitaxel‐induced cardiac inflammation and thus controls cellular inflammatory responses (Zhao et al. 2024). Silymarin mitigates chemotherapy‐induced cardiac injury, primarily by suppressing inflammatory pathways.

Alterations in the serum lipid profile often accompany Paclitaxel‐induced cardiotoxicity: increases in triglyceride (TG), LDL, and HDL, as well as total cholesterol (TC), and elevated LDH (a classic marker of cardiomyocyte damage) have been reported (Koneshamoorthy et al. 2022; Wang, Su, and Yin 2017). LDH is considered a classic biochemical marker of cardiomyocyte damage (Attanasio et al. 2024; Tonry et al. 2023). Clinical observations have reported cases of transient or severe hypertriglyceridemia during paclitaxel treatment, and therefore, TG monitoring is recommended. Furthermore, chemotherapy has been reported to disrupt lipid metabolism, leading to fluctuations in TC, TG, LDL, and HDL (Bhatnagar et al. 2022). Silymarin treatment significantly improved lipid profiles, with marked reductions in TC and LDL levels (approximately 55%) and decreases in TG levels (approximately 35–36%) in the silymarin‐treated group (Mohammadi et al. 2024). Silymarin was also found to lower TG levels and increase HDL in another meta‐analysis of patients with MASLD/NAFLD (Malik et al. 2024). Such antioxidant and anti‐inflammatory benefits might explain the improvements in lipid profile observed in our study (Zhao et al. 2024). Moreover, lipid levels in the silymarin‐treated group were very similar to those in the control group, suggesting that silymarin does not adversely affect lipid metabolism under normal physiological conditions. Thus, silymarin exhibits potent anti‐inflammatory properties and is among the best antioxidants in such cases. This effect aligns with meta‐analytic evidence showing silymarin's favorable influence on lipid and metabolic risk factors.

Under conditions of chemotherapy and inflammatory stress, an increase in HDL does not always reflect a beneficial outcome; during this process, HDL particles may lose their functional properties and transform into a “dysfunctional HDL” form (Rosenson et al. 2016). Therefore, the decrease in HDL observed in the silymarin group in our study can be interpreted as an indicator of possible functional improvement. Future evaluation of not only the amount of HDL but also its antioxidant and lipid‐efflux capacity is necessary (Besler et al. 2011).

Direct injury to cardiomyocytes caused by paclitaxel might lead to cardiotoxicity with the following mechanisms: oxidative stress, mitochondrial dysfunction, endothelial injury, and ferroptosis, while coronary vasospasm and histamine release mediated by mast cells respond to Cremophor EL‐containing formulations and are deemed the indirect cause (Gao et al. 2024; Tsao et al. 2022). The manifestations that were acute virtually included myocarditis, myocardial infarction, and even cardiac arrest, which were all related to the timing of paclitaxel infusion, with vasospastic and inflammatory pathways often pointed out as the causes (Johnson et al. 2024; Kim et al. 2024; Sh Ahmed et al. 2022). In line with these reports, our research detected structural alterations—vascular congestion, myofibrillar disarray, and mononuclear inflammatory infiltration—matching the currently accepted descriptions of chemotherapy‐related myocardial injury patterns (Gao et al. 2024). Silymarin treatment proved effective in curbing these pathological changes, which correlates with recent findings that silymarin offers cardioprotection through antioxidant and anti‐inflammatory pathways—namely, the suppression of NF‐κB signaling—and improves myocardial architecture in chemotherapy settings (İpek and Tunca 2023; Zhao et al. 2024).

Once P2X7R is activated, cytosolic Ca^2+^ levels are elevated, which in turn induces the release of pro‐inflammatory cytokines such as TNF‐α and IL‐1β, thereby amplifying myocardial injury through NF‐κB‐dependent mechanisms (Barberà‐Cremades et al. 2017; Giuliani et al. 2017). Further, in recent evidence from laboratory tests, it has been found that P2X7R overactivation contributes to mitochondria damage, generates reactive oxygen species, and initiates NLRP3 inflammasome in various tissues of the heart as well as the blood vessels, thus promoting cell death and consequently leading to tissue remodeling at an accelerated rate (Higashikuni et al. 2023; Shao et al.,2024). The work we conducted on paclitaxel‐induced cardiotoxicity supported the proposed mechanisms, as increased TNF‐α and IL‐1β levels coincided with activation of the P2X7R/NF‐κB signaling pathway. Silymarin was very effective in suppressing this signaling pathway by reducing TNF‐α and IL‐1β levels and by reducing the protein expression of P2X7R and NF‐κB p65. This result is consistent with earlier research on silymarin, which has shown that silymarin counteracts inflammation in liver and heart tissues by blocking the release of calcium ions and, consequently, cytokine release through the P2X7R/NF‐κB pathway (Zhao et al. 2024; Yakut et al. 2025). Combining all the findings, it can be concluded that silymarin likely exerts a powerful cardioprotective effect against paclitaxel‐induced harm by modulating the P2X7R/NF‐κB pathway. The P2X7R signaling might, as recent studies suggest, merge with the PI3K/Akt pathway. For instance, P2X7R activation has been shown to activate PI3K/Akt and downstream VEGF signaling, thereby fostering tumor cell survival and metabolic reprogramming (Pegoraro et al. 2021). Moreover, the PI3K/Akt pathway upregulates P2X7R expression in neuroblastoma cells, and P2X7R levels are drastically reduced when PI3K/Akt is inhibited (Gómez‐Villafuertes et al. 2015). Thus, it has been shown that the PI3K/Akt and P2X7R pathways reinforce each other and possibly escalate apoptotic and inflammatory processes. In our study, we found that paclitaxel not only increased PI3K p85α expression but also activated P2X7R/NF‐κB, suggesting that these pathways might cooperate in causing cardiac tissue damage. On the other hand, silymarin, by inhibiting signaling through both P2X7R/NF‐κB and PI3K, dismantled this connection, thereby unmasking a synchronized molecular mechanism underlying its cardioprotective properties.

Increased caspase‐3 activation and downregulation of the anti‐apoptotic protein Bcl‐2 are hallmarks of paclitaxel‐induced apoptosis in cardiac tissue, indicating the activation of intrinsic apoptotic pathways and the subsequent loss of cardiomyocytes. These changes were evident in our investigation, but silymarin treatment dramatically reversed them by lowering caspase‐3 levels and reestablishing Bcl‐2 expression. According to recent research, paclitaxel and other chemotherapeutic drugs induce mitochondrial apoptosis by activating NF‐κB signaling, dysregulating calcium homeostasis, and inducing oxidative stress (Gao et al. 2024). Consistent with our findings, silymarin has demonstrated strong anti‐apoptotic properties in models of drug‐induced organ damage. For instance, by inhibiting topoisomerase IIβ and reducing apoptotic cell death, silymarin mitigated doxorubicin‐mediated cardiotoxicity (İpek and Tunca 2023). Its capacity to inhibit caspase‐3 activation and improve Bcl‐2 levels in hepatic and cardiovascular models has also been highlighted in a thorough review (Zhao et al. 2024).

Although numerous preclinical studies support the antioxidant and anti‐inflammatory effects of silymarin, the literature reveals significant discrepancies. Some reviews question the direct improvement in endothelial dysfunction or atherosclerotic burden after silymarin treatment, arguing that the evidence remains quite limited (Kadoglou et al. 2022). Moreover, meta‐analyses of human studies showed improved glycemic and lipid profiles, whereas inflammatory markers and clinical cardiovascular endpoints remained essentially unchanged or only very slightly modified (Mohammadi et al. 2024). In addition, differences in dose, extract purity, treatment duration, and study populations complicate the interpretation of the results (Singh et al. 2023). So, our findings are consistent with most of the positive experimental data, but they should be viewed in light of this greater variability.

The data we have overall support a common mechanism in which paclitaxel causes heart damage via oxidative stress, mitochondrial dysfunction, and Ca^2+^ imbalance (Chang and Wang 2018; Gao et al. 2024). The activation of P2X7R by ROS leads to increased Ca^2+^ influx and cytokine release via NF‐κB, further fueling inflammation (Barberà‐Cremades et al. 2017; Giuliani et al. 2017; Higashikuni et al. 2023). Besides, paclitaxel activates PI3K signaling, which is a part of the apoptotic remodeling (Pegoraro et al. 2021; Gómez‐Villafuertes et al. 2015). Silymarin is a counteracting agent that limits oxidative stress (İpek and Tunca 2023), stops inflammation driven by NF‐κB (El‐Kot et al. 2023; Zhao et al. 2024), downregulates the signaling of P2X7R/NF‐κB, and it also plays a role in apoptosis regulation through caspase‐3 and Bcl‐2 (İpek and Tunca 2023; Zhao et al. 2024). All these activities together form a clear mechanistic basis for its cardioprotective effects in paclitaxel‐induced injury.

## 5 Limitations

This study has several limitations. First, only a single dose of silymarin was tested, preventing evaluation of dose–response relationships and limiting the ability to define an optimal therapeutic range. Second, although extensive biochemical, molecular, and histopathological analyses were performed, no cardiac functional assessments (e.g., electrocardiography, echocardiography, or hemodynamic measurements) were included; thus, it remains unclear whether the molecular improvements translate into preserved cardiac performance. Third, the study focused on an acute paclitaxel‐induced cardiotoxicity model, and potential long‐term or chronic remodeling effects were not evaluated. Finally, silymarin's well‐known limitation of poor oral bioavailability, which restricts its translational potential, was not directly addressed experimentally, and future studies should consider using enhanced‐bioavailability formulations to assess clinical relevance better. Future work incorporating multiple dosing regimens, functional imaging, and longer follow‐up will be essential to determine the durability and translational value of silymarin's cardioprotective effects.

## 6 Conclusion

Silymarin significantly reduced paclitaxel‐induced cardiotoxicity by attenuating oxidative stress, inflammation, and apoptosis in rat heart tissue. These findings highlight its promising therapeutic potential for mitigating chemotherapy‐related cardiac injury. However, further research—including functional cardiac assessments and dose‐range investigations—is required before clinical translation can be fully supported.

## Author Contributions

Conceptualization, T.T., A.K., S.Y.; methodology, T.T., A.K., and S.Y.; formal analysis, S.Y., and A.U.; investigation, C.Ç.; resources, T.T., S.Y., data curation, S.Y., T.T., writing – original draft preparation, T.T. and S.Y.; writing – review and editing, A.K., A.U., C.Ç.; project administration, T.T. All authors have read and agreed to the published version of this manuscript. The authors confirm that no paper mill or artificial intelligence was used.

## Funding

This research was funded by the Scientific Research Projects Unit of Bingöl University, Türkiye (project number BAP‐VF.2021.003).

## Ethics Statement

This study was approved by the Bingol University Animal Experiments Local Ethics Committee (28.06.2021–E.20008).

## Conflicts of Interest

The authors declare no conflicts of interest.

## Data Availability

The data that support the findings of this study are available from the corresponding author upon reasonable request.
